# Quantum optics with swift electrons

**DOI:** 10.1038/s41377-021-00530-9

**Published:** 2021-04-25

**Authors:** Nahid Talebi

**Affiliations:** Institute for Experimental and Applied Physics, Kiel University, 24118 Kiel, Germany

**Keywords:** Quantum optics, Nanophotonics and plasmonics

## Abstract

Stimulated and spontaneous interactions of electron wavepackets with optical near fields were explored with complementary techniques. In striking agreement with theory, scientists have demonstrated the dependence of spontaneous and stimulated quantum mechanical processes on the spatial distribution of optical modes.

The first quantum hypothesis reluctantly proposed by Max Planck, in an effort to describe black-body radiation in 1900, revolutionized the world of physics. The subsequent interpretation of the photoemission effect by Albert Einstein using Planck’s radiation theory initiated the birth of *quantum mechanics*—the phrase proposed by Max Born, Werner Heisenberg, and Wolfgang Pauli all at the University of Göttingen. In 1923, Louis De Broglie proposed that matters are waves, a contradictory description of *quantum mechanics*; thus, physics had to wait until the 1940s so that both quantum phenomena could be unified within the framework of quantum electrodynamics. Interestingly, a semiclassical theory, i.e., the theory that constitutes the interaction between matter waves, such as electron wavepackets and classical light (described by Maxwell’s equations), gained importance whenever spontaneous emission is negligible. However, the latter phenomena cannot be neglected when a few-photon process is considered: a situation that occurs during the interaction of an electron wavepacket with nanostructures to probe, for example, cathodoluminescence (CL) or electron energy-loss spectroscopy (EELS) responses^1^.

In parallel to quantum mechanical developments, the electron microscopy field emerged after the invention of the first electron microscope by Ernst Ruska and Max Knoll. Taking advantage of extremely short wavelengths of matter waves, such as ultrafast electron wavepackets, an ultimate resolution of a few tens of picometers is theoretically achievable. Everyday, experiments with electron microscopes, including recording diffraction patterns^2^ and performing holography^3^, highlight the unambiguous quantum nature of electrons as matter waves, thus verifying semiclassical theories. However, with the advent of ultrafast electron microscopy, which synchronizes laser excitations with pulsed electrons through the photoemission process, quantum electrodynamics experiments with an interplay between spontaneous and stimulated processes are becoming feasible. The field of photon-induced near-field electron microscopy (PINEM), invented by the late Nobel Laureate Ahmed Zewail^4^, involves stimulated processes instead of the spontaneous interactions of the EELS and CL spectroscopy techniques.

A recent publication by Liebtrau et al. reported exciting first steps toward performing quantum electrodynamic experiments with electron microscopy^5^. In an intelligent experiment, EELS, CL, and PINEM spectra were acquired and carefully analyzed. To conduct the experiments, a gold nanostar structure was used that, thanks to its sharp tips, hosts only single-mode dipolar resonances at its tips. By controlling the polarization of the laser beams in the PINEM experiment, it is possible to selectively excite certain tips. Since sharp tips do not sustain higher-order multipole resonances, both electrons and incident photons couple to the same mode when the light polarization and the propagation direction of electrons and incident photons allow for this. By carefully analyzing the spatial dependences of the EELS, PINEM, and CL rates, Liebtrau et al. showed the same spatial dependences for both stimulated and spontaneous processes in good agreement with theory, signifying the importance of the modal representation in describing the coupling efficiency between an external excitation and the system. Interestingly, theoretical predictions demonstrate similar spectral dependencies for both PINEM and CL that depend on the radiative nature of the mode and differ from EELS spectra. Comparing the CL and PINEM processes (Fig. 1a, b), this effect could be linked to the reciprocal nature of excitation and detection mechanisms in CL and PINEM experiments. Moreover, demanding that the laser intensity allows for multiple photon processes (Liebtrau et al. used a laser intensity of 20 MW/cm^2^), the electron wavepacket demonstrates a quantum walk on the energy ladder specified by photon energies (Fig. 1b)^6^. Moreover, nonlinear processes and Rabi oscillations could result in the complete depletion of the zero-loss peak as well^7^.

**Fig. 1:**
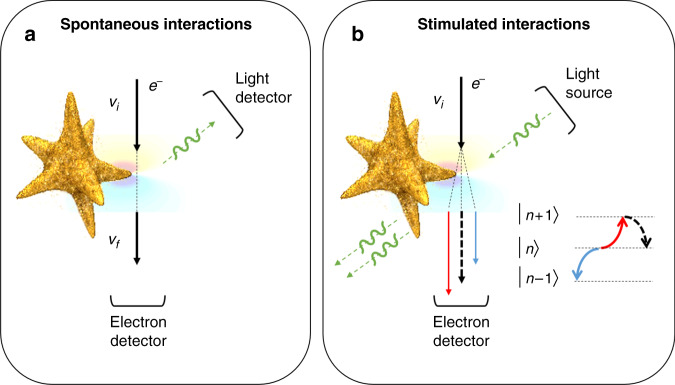
Spontaneous and stimulated processes in the interaction of electron beams with dipolar plasmonic resonances of a sharp tip are investigated using complementary approaches. **a** Conventional cathodoluminescence and electron energy-loss spectroscopy, where spontaneous interactions are responsible. **b** In a reciprocal approach to cathodoluminescence spectroscopy, the PINEM field addresses stimulated interactions. Depending on the laser intensity, multiphoton processes can lead to a complete depletion of the so-called zero-loss peak (black dashed arrow; Nanostar image, courtesy of Masoud Taleb and Maximilian Black, Kiel University).

In addition to the abovementioned experiments, Liebtrau and coworkers show that the maximum coupling between the electron and optical modes occurs for lower electron energies: indeed, an optimum coupling ensues for an electron at a kinetic energy of 3.5 keV. This behavior is due to the sharp tip apex (theoretically, a 3 nm curvature radius was considered), which leads to tightly bound evanescent tails of the plasmonic resonances so that the phase-matching condition could be met for only slow electron wavepackets^8^.

Technological developments in electron microscopy thus push the field toward performing quantum optics experiments and placing well-controlled electron beams next to single-photon emitters, as unique tools for performing such experiments. As perfectly demonstrated in the work by Liebtrau et al., the marvelous ability of electron microscopes to control the position of the beam and focus it into deep subwavelength spots allows quantum optics experiments to be performed on demand. This ability of electron microscopes circumvents the need to employ complicated schemes for controlling the position of single-photon emitters, such as quantum dots with respect to nanostructures. Therefore, by introducing an external laser field and controlling its intensity, it becomes possible to switch between spontaneous and stimulated processes.

CL and EELS hence both work within spontaneous interaction processes, where only single-photon exchanges are normally possible; though it was recently demonstrated that using slow electrons interacting with graphene, one could expect multiphoton processes as well^9^. New experiments that compare CL, EELS, and PINEM in graphene enabling multiphoton spontaneous processes could add a novel perspective in this regard. Moreover, although theoretical works predict an optimum energy for the electron that enables a strong coupling to the field, PINEM experiments with slow electrons within the range of intermediate electron energies reachable by point-projection microscopes, and scanning electron microscopes should be performed to test, for example, the validity of nonrecoil approximations^10^.

## References

[1] Polman A, Kociak M, García de Abajo FJ (2019). Electron-beam spectroscopy for nanophotonics. Nat. Mater..

[2] Bach R (2013). Controlled double-slit electron diffraction. N. J. Phys..

[3] Tonomura A (1987). Applications of electron holography. Rev. Mod. Phys..

[4] Barwick B, Flannigan DJ, Zewail AH (2009). Photon-induced near-field electron microscopy. Nature.

[5] Liebtrau M (2021). Spontaneous and stimulated electron-photon interactions in nanoscale plasmonic near fields. Light: Science & Applications.

[6] Park ST, Lin M, Zewail AH (2010). Photon-induced near-field electron microscopy (PINEM): theoretical and experimental. N. J. Phys..

[7] Feist A (2015). Quantum coherent optical phase modulation in an ultrafast transmission electron microscope. Nature.

[8] Talebi N (2020). Strong interaction of slow electrons with near-field light visited from first principles. Phys. Rev. Lett..

[9] Cox JD, García de Abajo FJ (2020). Nonlinear interactions between free electrons and nanographenes. Nano Lett..

[10] Talebi N (2018). Electron-light interactions beyond the adiabatic approximation: recoil engineering and spectral interferometry. Adv. Phys. X.

